# Prevalence of widespread pain and associations with work status: a population study

**DOI:** 10.1186/1471-2474-9-102

**Published:** 2008-07-15

**Authors:** Björn Gerdle, Jonas Björk, Lars Cöster, KG Henriksson, Chris Henriksson, Ann Bengtsson

**Affiliations:** 1 Department of Rehabilitation Medicine, Faculty of Health Sciences, Linköping University, Linköping, Sweden; 2 Department of Rheumatology, Faculty of Health Sciences, Linköping University, Linköping, Sweden; 3 Department of Occupational Therapy, Faculty of Health Sciences, Linköping University, Linköping, Sweden; 4 Pain and Rehabilitation Centre, University Hospital, Linköping, Sweden; 5 Competence Centre for Clinical Research, Lund University Hospital, Lund, Sweden

## Abstract

**Background:**

This population study based on a representative sample from a Swedish county investigates the prevalence, duration, and determinants of widespread pain (WSP) in the population using two constructs and estimates how WSP affects work status. In addition, this study investigates the prevalence of widespread pain and its relationship to pain intensity, gender, age, income, work status, citizenship, civil status, urban residence, and health care seeking.

**Methods:**

A cross-sectional survey using a postal questionnaire was sent to a representative sample (n = 9952) of the target population (284,073 people, 18–74 years) in a county (Östergötland) in the southern Sweden. The questionnaire was mailed and followed by two postal reminders when necessary.

**Results:**

The participation rate was 76.7% (n = 7637); the non-participants were on the average younger, earned less money, and male. Women had higher prevalences of pain in 10 different predetermined anatomical regions. WSP was generally chronic (90–94%) and depending on definition of WSP the prevalence varied between 4.8–7.4% in the population. Women had significantly higher prevalence of WSP than men and the age effect appeared to be stronger in women than in men. WSP was a significant negative factor – together with age 50–64 years, low annual income, and non-Nordic citizen – for work status in the community and in the group with chronic pain. Chronic pain but not the spreading of pain was related to health care seeking in the population.

**Conclusion:**

This study confirms earlier studies that report high prevalences of widespread pain in the population and especially among females and with increasing age. Widespread pain is associated with prominent effects on work status.

## Background

Often, subjects with chronic pain have pain in several anatomical regions. Chronic musculoskeletal pain represents a continuum with chronic widespread pain (WSP) including fibromyalgia syndrome (FMS) as the most severe clinical manifestations [1-3]. The prognosis of WSP appears to be poor according to some studies [4-6]; e.g., in a population cohort WSP had a poor prognosis with respect to resolution at least in a 12-year perspective [5]. But other studies have reported results that question the constancy of chronic WSP [7,8].

In a study of occupationally active female home care personnel, a prevalence of 14.4% WSP was reported according to the definition as defined by the FMS criteria of the American College of Rheumatology (ACR) [9]. In other Swedish studies, Lindell et al. reported a prevalence of 4.2% [7] while Bergman et al. reported a prevalence of 11.4% [10]. In a population-based study from Oslo, Norway, a prevalence of 10% of generalised pain was found among women [11]. Studies outside Scandinavia report figures between 4.7–11.2% [12-16]. In one study, an absence of chronic WSP was found in Pima Indians in Arizona, USA [17]. To summarize relatively large differences in prevalence of WSP in the community have been reported.

Different definitions of WSP have been used in the literature, e.g.,: 1) pain at more than three locations in both the upper and lower half of the body [4]; 2) pain in at least two sections of two contra lateral limbs and in the axial skeleton (i.e., the Manchester definition) [13]; and 3) pain is considered widespread when all of the following are present: pain in the left side of the body, pain in the right side of the body, pain above the waist, and pain below the waist; axial skeletal pain (cervical spine or anterior chest or thoracic spine or low back) must be present (i.e., the ACR definition of chronic WSP as part of the classification criteria for FMS) [18]. Furthermore, the ACR definition of chronic WSP, has apparently been interpreted in different ways [14,19]. The different definitions of WSP might partly explain differences in prevalence and prognosis reported in the literature. The ACR and Manchester definitions of WSP are the most commonly applied definitions and there is a need to compare the prevalence figures obtained using these two definitions.

In the literature there are several indications that certain sociodemographic factors are associated with WSP. Women in most studies report higher prevalences of WSP than men and the prevalence also increases with age [10,12,14,16,20]. How much more common the condition is in women than men differ relatively prominently between studies; the prevalence was nearly 5 times higher in women than in men in Israel while studies from UK report figures lower than 2 times [12,21]. Bergman et al. reported – besides age and gender – that WSP was associated with being an immigrant, living in a socially comprised housing area and being an assistant non-manual lower level employee or manual worker [10]. Subjects with WSP significantly more often also had a lower education level [10]. Due to the fact that several of these factors most likely are intercorrelated it is important to understand how they relate to WSP in a more comprehensive (multivariate) context when planning for instance prevention, health care and social security systems.

The literature summarised above might indicate that WSP has great impact on working life, health care and social security systems. The present study is part of a larger population-based project concerning prevalence of pain in a county in southern Sweden. In the first study we reported a prevalence of 54% for chronic pain (i.e., pain > 3 months and irrespective of pain intensity) [22]. We also reported considerable effects of chronic pain (especially intensive and frequent pain) on health care seeking and work status [22]. The correlation between WSP and work status is seldom investigated even though some studies have reported negative consequences with respect to work status [4,23]. Buskila et al. reported that the group with WSP in comparison to chronic regional pain had more visits to their physicians, were more frequently referred to specialists, and used more anti-inflammatory and analgesic drugs [16]. WSP was also associated with significantly increased prevalences of other symptoms and disorders [12,13,21,24] and it is reasonable to suspect that such co-morbidity contributes to the increased use of health care in WSP. The question arises if the spreading of pain is associated with increased work disability and health care compared to chronic pain.

Based on the review of the literature we hypothesised that: a) WSP is common in the population, b) considerable differences in prevalences of WSP exist between the ACR and Manchester definitions, c) WSP is more common in women than in men and increases with age d) not only chronic pain but also spreading of pain (i.e., WSP) is associated with increased consumption of health care and work disability.

Hence, the main aims of this population-based cross-sectional study are as follows:

• Investigate the prevalence of WSP in the population using two common constructs. Within this aim we investigated the prevalence of pain in ten different anatomical regions.

• Multivariately analyse the impacts of gender, age, civil status, income, and citizenship upon the prevalence of WSP.

• Investigate to what extent WSP interacts with work status and health care seeking.

## Methods

### Design

The study was a cross sectional survey using a postal questionnaire to collect data from a representative sample of the population (18–74 years) from a county (Östergötland) in the southern part of Sweden [22]. The target population was 284,073 persons in the age group 18–74 years. A representative sample of 9952 subjects was selected from the register of Statistics Sweden (SCB). In September 1999, a questionnaire was mailed and was followed by 2 postal reminders if necessary. The first reminder was mailed after approximately 4 weeks and the second reminder approximately 3 weeks later.

The Ethics Research Committee, Linköping University, Sweden, approved the study.

### Questionnaire

The questionnaire contained the following items:

#### Pain measures

**Figure 1:**
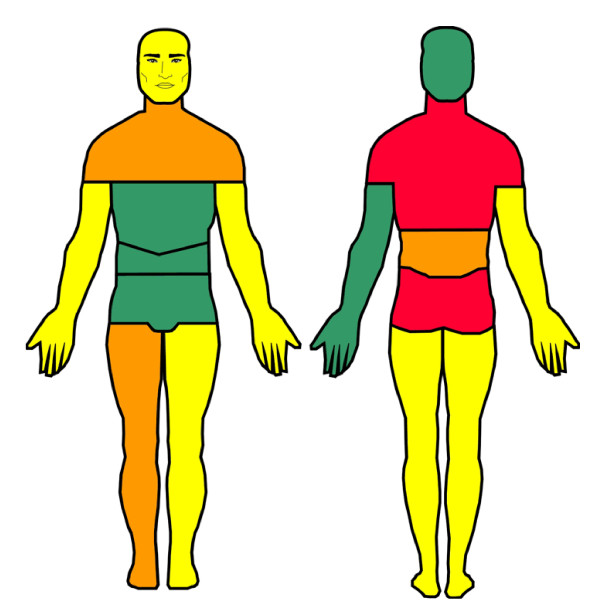
Prevalence of pain during previous week in 17 pre-defined anatomic regions (n = 7637): Green: 0–9.9%, Yellow: 10.0–14.9%; Orange: 15.0–19.9%; and Red: ≥ 20.0%.

1) prevalence of pain in the previous week in 17 predefined anatomical regions (indicated on a drawing; figure 1);

2) duration of persistent pain *(more than three months or less than three months*);

3) whether the responder had sought health care for the pain *(Yes or No)*;

4) types of health care (the following alternatives not excluding each other's were presented: *district doctor/health centre, clinic of a hospital, private doctor, company doctor, paramedical personnel (physiotherapist, chiropractor, naprapathy, acupuncture) and other)*;

#### Work measures

5) work situation (alternatives not excluding each other's: *work, sick-leave, studies, parental leave, looking for work, disability pension, retirement pension, and other*);

6) work status *(0 hours per week, 1–15 hours/w, 16–24 hours/w, 25–34 hours/week or ≥ 35 hours/w)*,

7) amount of sick leave or temporary disability pension (*0%, 1–25%, 50%, 75% or 100%);*

#### Sociodemographic measures

8) gender *(male or female)*;

9) nationality *(country; this information was dichotomized into Nordic citizen (Sweden, Norway, Denmark, Finland and Iceland) or non-Nordic citizen (i.e., other countries))*;

10) civil status *(married/cohabiting or single)*;

11) age *(years)*;

12) income *(SEK per year)*;

13) municipality *(city or in the country)*.

In the questionnaire (5 pages), no definition of pain was made and thus the subjective perception of the concept *pain *was reported. Persistence of pain and health care utilization were not mentioned for each pain site separately due to the risk for recall bias. The 17 anatomical areas of the drawing in the questionnaire (Figure 1) were grouped into 10 more adequate anatomical regions (Table 1) that were used in the multivariate analysis. The following regions were identified: head, right arm, left arm, right leg, left leg, neck, shoulders, and thoracic spine (in the following labeled upper back), chest anterior part, abdomen, low back and pelvis anterior part. Based on these 10 anatomical regions, two new variables intended to reflect widespread pain (WSP) were developed:

**Table 1:** Prevalence (%) of pain in previous week in the 10 grouped anatomic regions and proportion with number of anatomical regions ≥ 3 out of the 10 anatomical regions stratified by gender (n = 7637).

Region	Men	Women	All
Head	10.1	21.8	16.2
Upper back (i.e., neck, shoulders and thoracic spine)	33.2	44.8	39.3
Right arm	13.2	20.4	17.0
Left arm	11.5	16.8	14.3
Chest anterior part	5.6	6.8	6.2
Abdomen	3.8	6.1	5.0
Pelvis, anterior part	5.4	10.3	8.0
Low back	29.1	36.6	33.0
Right leg	16.6	22.4	19.6
Left leg	16.8	20.0	18.5
Proportion (%) with number of anatomical regions with pain ≥ 3 (out of the 10 anatomical regions above)	22.3	33.9	28.4

• Spinal pain in any part and pain in the four extremities formed a binary variable intended to reflect the widespread pain definition of ACR (labelled *WSP4limbs*);

• *WSPconlimbs *was defined as spinal pain and contra-lateral limb pain and intended to reflect the Manchester definition of WSP [13].

We also determined *the number of the ten anatomical regions with pain *(possible range: 0–10). In the regressions, this variable was dichotomised (0–2 versus 3–10).

### Statistics

All descriptive statistics were obtained using the statistical package SPSS for Windows (version 12.0.1; SPSS Inc.). Characteristics of the participants as well as of the non-participants were generally presented as proportions (binary variables) or as medians and 10^th^–90^th ^percentiles (counts or continuous variables). Multivariate analyses were conducted in EGRET for Windows (version 2.0; Cytel Software Corp.) with the following outcome variables: 1) the widespread pain indicators, and 2) the prevalence of working at least 25 hours a week. The association between each of these outcome variables and gender, age, annual income, civil status, urban residence, citizenship, and sick leave or pre-retirement were modelled using Cox proportional hazards model with a constant risk period of unit length [25]. Cox regression was used in order to estimate prevalence ratios (PRs) instead of prevalence odds ratios (obtained in logistic regression), which are more difficult to interpret. To estimate the prevalence for the reference category of the multivariate model, we used the corresponding parametric regression model for survival data based on the exponential distribution. The Cox proportional hazards model is multiplicative, implying that if the (pain) PR for females versus males is 1.29 and the PR for the age group 50–64 versus the age group 18–29 is 1.23, then the PR for females of the age group 50–64 versus males of the age group 18–29 is 1.29·1.23 ≈ 1.59. Two-sided p-values below 0.05 were considered as statistically significant.

## Results

### Participation rate

The participation rate was 76.7% (n = 7637). Background characteristics of the participants as well as of the non-participants have been presented elsewhere [22]; the non-participants were on average younger, earned less, and were more often males.

### Pain in different anatomical regions

Pain in at least one anatomic area in the previous week was reported by 63.2% participants. According to the 10 anatomic regions, the upper back (39.3%), and low back (33.0%) had the highest prevalences (Table 1). Women had higher prevalences of pain than men in each of the 10 grouped anatomic regions (Table 1). Nearly one third of the population had pain in at least 3 out of 10 anatomical regions (Table 1).

### Widespread pain

When applying the ARC criteria for WSP (i.e., *WSP4limbs*) an overall prevalence of 4.8% was found (Table 2). Using *WSPconlimbs *(i.e., the Manchester definition) resulted in a higher overall prevalence 7.4%. The proportion of *chronic *pain (pain that has lasted more than 3 months) among subjects with widespread pain according to the two widespread pain variables varied between 90 and 94%. The associations between gender and age and the two widespread pain indicators were apparent (Table 2).

**Table 2:** Prevalence of widespread pain recent week (%) according to two indicators of WSP (see text), stratified by age and gender (n = 7637).

Variables	Age group	All	Men	Women
WSP*4limbs*	All	4.8	3.0	6.5
	-29	1.6	2.1	1.2
	30 – 49	4.1	2.1	5.8
	50 – 64	7.0	3.9	9.9
	65-	6.7	4.8	8.3

*WSPconlimbs*	All	7.4	4.9	9.7
	-29	2.4	2.8	2.0
	30 – 49	6.3	3.7	8.5
	50 – 64	11.3	6.7	15.6
	65-	9.1	6.9	11.0

Widespread pain according to *WSP4limbs *was in the multivariate analysis significantly associated with gender, age, sick leave or pre-retirement, annual income, and citizenship, whereas civil status and urban residence were not significant (Table 3). Females had a higher prevalence of widespread pain than males except in the youngest age group (<30 years). The effect of age on *WSP4limbs *thus seemed stronger among females than among males. The same general pattern as for *WSP4limbs *emerged when widespread pain was measured as *WSPconlimbs *(Table 3).

**Table 3:** Cox regression of the association between widespread pain recent week, defined as WSP *4limbs*, and WSP*conlimbs*, respectively and gender, age, sick leave or pre-retirement, annual income, and citizenship (n = 7637).^a^

	WSP*4limbs*		WSP*conlimbs*	
Explanatory variable	Prevalence ratio (PR)	95% confidence interval	Prevalence ratio (PR)	95% confidence interval

Reference category	1.0^b^	-	1.0^c^	-
Age effect among females				
Age				
< 30	0.51	0.22 – 1.16	0.65	0.33 – 1.27
30–49	2.56	1.47 – 4.44	2.83	1.76– 4.54
50–64	3.80	2.18 – 6.63	4.49	2.79 – 7.21
65-	3.99	2.23 – 7.13	3.92	2.37 – 6.50
Age effect among males				
Age				
30–49	1.23	0.65 – 2.31	1.50	0.89 – 2.54
50–64	1.81	0.98 – 3.34	2.21	1.32 – 3.69
65-	2.83	1.47 – 5.44	2.84	1.63 – 4.96
Sick leave/pre-retirement	2.76	2.13 – 3.58	2.47	2.00 – 3.05
Income below median	1.79	1.41 – 2.28	1.46	1.21 – 1.76
Non-Nordic citizen	2.40	1.56 – 3.71	1.86	1.24 – 2.78

^a ^Civil status and urban residence had no significant impact on the pain prevalence and did not act as confounders and were thus omitted from the regression model.

^b ^The model-based estimate of the prevalence for the reference category, i.e., for male Nordic-citizens below the age of 30, not on sick leave or pre-retired, and with an income above median was 1.2% (95% confidence interval 0.7 – 2.1%).

^c ^The model-based estimate of the prevalence for the reference category, i.e., for male Nordic-citizens below the age of 30, not on sick leave or pre-retired, and with an income above median was 2.0% (95% confidence interval 1.3 – 3.2%).

### Widespread pain and work status

Both the number of anatomical regions with pain ≥ 3 and fulfilling *WSP4limbs *had significant impact on the work status *in the population below the age of 65 years *(Table 4). The isolated effects of the number of anatomical regions with pain ≥ 3 and WSP*4limbs *were 12% and 25%, respectively, reduction in the prevalence of work activity, here defined as working at least 25 hours/week. For those with a high the number of anatomical regions with pain (i.e., ≥ 3–10 out of 10) below the age of 65 years (n = 1883), only 16% also fulfilled the criteria of *WSP4limbs*. When both these were present, about 30% reduction in work activity was noted. Using WSP*conlimbs *instead of WSP*4limbs *as WSP indicator yielded similar estimated reductions in the prevalence of work activity (WSP*conlimbs*: PR = 0.81, 95% CI 0.70 – 0.95; anatomical regions with pain = 3: PR = 0.88, 95% CI 0.82 – 0.96; not in tables). The most prominent effect was linked to income, but age 50–64 years and citizenship also had marked effects.

**Table 4:** Cox regression of the association between work status (i.e., the prevalence of working at least 25 hours a week) and number of anatomical regions with pain ≥ 3, WSP*4limbs*, age, annual income, and citizenship in *the group below the age of 65 *(n = 5780).^a,b^.

Explanatory variable	Prevalence ratio (PR)	95% confidence interval
Reference category^c^	1.0	-
Number of anatomical regions with pain ≥ 3	0.88	0.81 – 0.94
*WSP4limbs*	0.75	0.62 – 0.92
Age		
30–49	0.99	0.90 – 1.09
50–64	0.77	0.70 – 0.86
Income below median	0.62	0.57 – 0.66
Non-Nordic citizen	0.74	0.57 – 0.95

^a ^Acute or chronic (but not necessarily widespread, defined as number of anatomical regions with pain ≥ 3 or WSP*4limbs*) pain, gender, civil status, and rural residence had no significant impact on the prevalence of working at least 25 hours a week and did not act as confounders and were thus omitted from the regression model.

^b ^Full-time students and persons on full-time parental leave were excluded (n = 803).

^c ^The model-based estimate of the prevalence for the reference category, i.e., for Nordic citizens without widespread pain, defined as number of anatomical regions with pain ≥ 3 or *WSP4limbs*, below the age of 30, and an income above median was 96.9% (95% confidence interval 88.3% – 100%).

In the group of *subjects with chronic pain*, pain intensity, but not citizenship, had influence upon work status (Table 5). Thus, *WSP4limbs*, pain intensity, age 50–64 years, and income were significant determinants of work status in the chronic pain group. An almost identical effect estimate for WSP was obtained when WSP*conlimbs *instead of WSP*4limbs *was used (PR 0.74, 95% CI 0.59 – 0.93; not in tables).

**Table 5:** Cox regression of the association between work status (i.e., the prevalence of working at least 25 hours a week) and *WSP4limbs*, intensity of pain, age, and annual income in the group with *chronic pain below the age of 65 *(n = 1771 with complete data).^a,b^.

Explanatory variable	Prevalence ratio (PR)	95% confidence interval
Reference category^c^	1.0	-
WSP*4limbs*	0.74	0.56 – 0.99
Unbearable/severe pain intensity	0.80	0.69 – 0.92
Age		
30–49	0.94	0.79 – 1.13
50–64	0.76	0.63 – 0.91
Income below median	0.58	0.51 – 0.66

^a ^Number of anatomical regions with pain ≥ 3, frequency of pain, gender, civil status, rural residence, and citizenship had no significant impact on the prevalence of working at least 25 hours a week and did not act as confounders and were thus omitted from the regression model.

^b ^Full-time students and persons on full-time parental leave were excluded (n = 321).

^c ^The model-based estimate of the prevalence for the reference category, i.e., for persons with chronic (but not widespread, defined as *WSP4limbs*) pain of at most moderate intensity, below the age of 30, and an income above median was 100% (95% confidence interval 85.6% – 100%).

### Widespread pain and health care seeking

In our earlier study based on the same sample from the population, we reported that age, pain frequency, intensity, and sick-leave/pre-retirement were significant factors interacting with health care seeking in the group chronic pain. After adjusting for the factors that were significant determinants of the prevalence of health-care seeking in the chronic pain group (age, pain frequency and intensity, and sick-leave/pre-retirement), the different widespread variables all had PRs close to unity (WSP*4limbs*: PR = 0.95, 95% CI 0.77–1.16, WSP*conlimbs*: PR = 0.97, 95% CI 0.82–1.15, WSP*index *= 3: PR = 1.02, 95% CI 0.92–1.14; not in tables).

## Discussion

### Major findings

The following are the major results that will be discussed in this section:

a) WSP generally was chronic and depending on definition of WSP the prevalence varied between 4.8–7.4% in the population.

b) Women had generally significantly higher prevalence of WSP than men and the age effect appeared to be stronger in women than in men.

c) WSP was a significant negative factor – together with age 50–64 years, low annual income, and non-Nordic citizen – for work status in the community and in the group with chronic pain.

d) Chronic pain but not the spreading of pain (i.e., WSP) was related to health care seeking in the population.

### Localised pain

The majority of the subjects reported pain from any of the ten anatomical regions for the previous 7 days; i.e., pain is obviously a very common experience in the population and part of many people's every day life. The relatively high prevalences of pain in the head, upper back and lower back are in agreement with other studies [26-29]. But it is difficult to compare different studies due to factors such as how a certain anatomical region was defined, the length of the prevalence period, definition of pain, data collection techniques, etc. For the 10 anatomic regions, women had higher prevalences of pain than men. Such gender difference has also been found in other studies [20,26,28-30]. In some studies, the anatomical region with highest prevalence has differed between the sexes [31], but this was not found in the present study.

### Widespread pain

#### WSP is generally chronic and increases with age

Based on the present study, it can be concluded that WSP generally was chronic and increases with age. These observations are probably due to the fact that most of the widespread pain conditions start as local or regional pain syndromes often in the neck-shoulder region and that it takes considerable time before the generalization occurs [9,32-37]. Another factor is that chronic WSP generally is a non-fatal condition leading to accumulation of cases in older age groups [38].

#### Prevalence of WSP

Most studies in the literature report – as in the present study – relatively high prevalences of WSP in the community of different countries – between 4.2–11.4% [7,10-16]. The definitions of WSP used in these studies vary but are usually related to the ACR or the Manchester definitions. One important result from the present study is that the definition of WSP chosen will lead to relatively marked differences in prevalence of WSP. Using the Manchester definition (i.e., WSP*conlimbs*) markedly higher prevalence was found compared to the ACR definition; in all subjects taken together 7.4% versus 4.8%. It was beyond the scope of this study to analyze which of the two WSP variables that was most valid.

In clinical practise the first step in the diagnosis of FMS is to determine if WSP is present or not (i.e., asking the patient and/or determined from different types of drawings). When WSP is present, a tender point examination will follow in order to determine if hyperalgesia exist and herby the ACR criteria of FMS are fulfilled. Hence a part of the variability between studies of the prevalence of FMS might be due to the exact WSP definition applied. Thus, both in clinical practise and research studies of WSP including FMS it is very important to relate the obtained figures to the widespread pain definition.

#### Gender

Women had generally higher prevalence of WSP than men, a finding that agrees with other studies [12,21]. The reasons for these differences between genders are unclear [38] but are not due to sex hormonal factors according to one study [39]. How much more common the condition is in women than men differ between studies; ratios between approximately 5 and 2 times exist in the literature [12,21]. In all subjects taken together, the ratios were in agreement with the latter figure for the two WSP definitions. Furthermore, the ratio between women and men is markedly lower than for the diagnosis FMS in the clinic and in the population [23,40]. This might partly be due to gender differences in pressure pain thresholds; men appear to have higher pain thresholds than healthy women according to several studies although such investigations can be influenced by contextual and distress factors [40,41]. Other alternatives are that hyperalgesia at tenderpoint examination is more prevalent in women and/or that only a selection of subjects with WSP also have the diagnosis FMS [7,42].

Another interesting finding of this study was that the prevalence of WSP showed different age relations for men and women. For both WSP variables we found that the age effect appeared stronger among women than among men. We have no simple explanation for these findings and according to our judgement it is reasonable to expect a multitude of variables both related to biological (sex) and social (gender) factors explaining this observation.

#### Immigrants

Agreeing with Bergman et al., we found higher prevalences of WSP in non-Nordic subjects than in Nordic subjects [10]. Several studies from Sweden show that immigrants have more musculoskeletal disorders and are over represented among those who are pre-retired [43-46]. Some experimental studies have shown ethno-cultural influences in aspects of pain, but there appears not to be a consistent pattern [47-50]. Many immigrants have fled from war and other traumatizing situations associated in different ways with high incidence of pain. There might also be difficulties to get an employment in Sweden that corresponds to the level of education; i.e., an overrepresentation in lower socioeconomic groups that in turn consist of occupations with overrepresentation of risk factors for pain (see below). Against our result could be argued that the proportion of non-Nordic subjects was low and the cross-sectional design of the study limit conclusions regarding cause-effects.

#### Level of income

We found that low annual income was significantly linked to WSP. There are studies that have reported associations between blue collar work, low income, or low level of education and chronic musculoskeletal pain [51-55]. The annual income variable was chosen in order to reflect the socio-economic position, but it can in retrospect be argued that this variable was too unspecific when trying to reflect the socio-economic position. The effect of income differs across countries due to factors such as the construction of the social security system. A low annual income could also reflect difficult working conditions. Some pain conditions are work-related; there is consensus or near consensus that musculoskeletal pain conditions of neck, shoulders, and low back are causally related to certain ergonomic or physical factors in the work environment [56,57]. Psychosocial factors are other recognized risk factors for such pain conditions and there are reports in the literature that there might be an interaction effect between ergonomic and psychosocial factors at the work place [56-62]. Based on a retrospective analysis in accordance with other studies, Larsson and Balogh reported that FMS generally debuted as a local or regional pain condition [32]. Moreover, subjects that later developed FMS at debut of pain were overrepresented in highly repetitive work tasks, which are known as risk factors for neck-shoulder pain [32]. Whether such mechanisms also are present for the whole group of WSP is unknown at present.

### WSP and work status

WSP and number of anatomical regions with pain ≥ 3 had significant impact on the work status *in the population*. If anything, the effect of WSP was somewhat stronger than the effect of number of anatomical regions with pain ≥ 3. When both these circumstances were fulfilled, marked effects were obtained (about 30% reduction in work activity). In our earlier report from the same cohort concerning chronic pain, we reported that chronic pain but not acute pain was significantly related to the work status in the community [22]. The effect of chronic pain on work status was equal to that of number of anatomical regions with pain ≥ 3. We also identified age 50–64 years, low annual income, and non-Nordic citizen as other determinants of low work status. Thus, this cross-sectional study indicates that a complex pattern of chronic pain, spreading of pain and factors related to age, income, and citizenship must be taken into consideration when analyzing mechanisms related to work and sick leave in the Swedish society.

In our previous analysis of chronic pain, we found that pain intensity and to some extent (marginally significant) frequency of pain together with age 50–64 years and low annual income influenced work status *in the chronic pain group *< 65 years [22]. In the present analysis of the chronic pain group, now also including WSP, the importance of pain intensity remained, but WSP was also an independent determinant of work status.

In conclusion our results clearly show that WSP together with other factors are significant negative factors both for work status in the community and within the group of the population with chronic pain. These results are in agreement with studies of patients with FMS, although there is some variability between countries, a substantial part cannot continue to work [63]. Such results implies substantial personal and societal economic consequences.

### Health care seeking in WSP

In our recent study of chronic pain in the community (the same cohort as in the present study) [22], we confirmed other reports that pain and pain intensity are associated with considerable amount of *health care seeking *[64-68]. Based on the present study, it can be concluded that the spreading of pain does not significantly contribute to the model presented of health care seeking, which identified pain frequency and intensity, age, and sick-leave/pre-retirement as significant regressors [22].

### Strong points and Study limitations

The facts that we have investigated how WSP and other factors influence work status are advantages when compared to several other community-based studies. This study also uses a large sample size. The statistical uncertainty in the estimated strength of the associations is therefore generally low. The participation rate is of more concern and could produce bias if participation is associated both with WSP and e.g. work status. The validity of the questionnaire should also be considered in future studies. Another limitation was the cross-sectional setup that limits definite conclusions regarding cause-effects for e.g. the association between WSP and work status and level of income.

## Conclusion

The present results confirm earlier studies that report high prevalences of widespread pain in the population and especially among females and with increasing age. The results of this study also show that widespread pain is associated with prominent effects on work status.

## Competing interests

The authors declare that they have no competing interests.

## Authors' contributions

BG wrote the main part of the first version of the manuscript and made revisions after comments from the other authors. JB carried out the regression analyses and had comments on the different versions of the manuscript. LC participated in the design of the study and construction of the questionnaire and had comments on the different versions of the manuscript. KGH participated in the design of the study and had comments on the different versions of the manuscript. CH participated in the design of the study and wrote parts of the methods section. AB participated in the design of the study and construction of the questionnaire and had comments on the different versions of the manuscript. All authors read and approved the final manuscript.

## Pre-publication history

The pre-publication history for this paper can be accessed here:


